# 

**Published:** 1859-05

**Authors:** 


### CONTENTS.

### ORIGINAL COMMUNICATIONS.

PAGE.

ARTICLE I.—Spermatorrhœa.—A report by T. A. Means, M. D., of Memphis, Tenn., read before the Medical Association of that City,............... 517

ARTICLE II.—Sulphate of Cinchonia. By Robert Battly, M. D., Rome, Georgia,.............................................................. 531

ARTICLE III.—Remarks on the Modus Operandi of Calomel. By Hayden Coe, M. D. of Atlanta, Ga.,.................................................. 533

### SELECTIONS.

Puerperal Phlebitis—Purulent Infection—with remarks,........................ 539

London Correspondence,.................................................. 548

Cases Illustrative of Criminal Abortion,.................................. 553

On the comparative Influence of the Male and Female Parent upon the Progeny,.............................................................. 557

Cases of sudden Death attributed to Imprudent Bathing,........................ 561

Kameela—The New Anthelmintic,.......................................... 563

On a case of Prolapsus Uteri, cured without Operation or the necessity of Wearing a Pessary,....................................................... 565

### EDITORIAL AND MISCELLANEOUS.

Abstract of the proceedings of the Medical Association of the State of Georgia,................................................................ 567

Thomas A. Means, M. D,................................................ 576

The Treatment of Cancer.—Cancer Quacks,................................ 577

Application of Glycerine in Variola,........................................ 580

